# Progressive episodes of recurrent syncope due to postural orthostatic tachycardia syndrome truncated by paroxetine: a case report

**DOI:** 10.3389/fpsyt.2025.1576740

**Published:** 2025-05-16

**Authors:** Hai Liang, Chen Liang, Lihua Chen, Jiayang Fang, Yongxin Yi, Yidong Gao, Xiang Huang, Xi Chen, Ting Liu, Wei Pan, Lufeng Yin

**Affiliations:** ^1^ Department of Neurology, The Third Affiliated People’s Hospital of Fujian University of Traditional Chinese Medicine, Fuzhou, China; ^2^ Department of Electrophysiology, The Third Affiliated People’s Hospital of Fujian University of Traditional Chinese Medicine, Fuzhou, China

**Keywords:** postural orthostatic tachycardia syndrome, paroxetine, recurrent syncope, anxiety disorders, norepinephrine transporter proteins

## Abstract

Postural orthostatic tachycardia syndrome (POTS) is characterized by an increased heart rate upon standing, resulting from abnormal autonomic responses that trigger symptoms when transitioning to an upright position. This syndrome predominantly manifests in late adolescence and early adulthood, with a higher prevalence observed in women. It is commonly triggered by viral infections, pregnancy, surgical procedures, or significant psychological stress. The condition presents with a wide range of symptoms, and the precise etiology of which remains unidentified. A 17-year-old woman with recurrent syncope was admitted to the hospital multiple times. Symptoms resolved rapidly following the initiation of paroxetine, a selective serotonin reuptake inhibitor (SSRI). Preliminary analysis indicated a potential shared pathophysiological basis between POTS and anxiety disorders, with norepinephrine transporter proteins emerging as a significant therapeutic target for both conditions.

## Introduction

Postural orthostatic tachycardia syndrome (POTS) is a form of orthostatic intolerance resulting from dysautonomia, typically presenting with pre-syncopal symptoms, such as dizziness, palpitations, tremor, generalized weakness, blurred vision, exercise intolerance, and fatigue particularly in a standing position. POTS is one of the most prevalent autonomic nervous system (ANS) disorders, characterized by excessive sinus tachycardia upon standing. It can lead to significant functional impairment, typically limiting an individual’s ability to work or attend school. As a result, the condition often goes undiagnosed for prolonged periods and is frequently misinterpreted as an anxiety disorder. Misdiagnoses can be attributed to the association of anxiety with symptoms, such as tachycardia, palpitations, and dizziness (1). Symptoms of POTS usually occur while standing, especially after rapid standing, whereas symptoms of anxiety may occur without an obvious trigger and last longer. POTS symptoms are typically posture-dependent, while anxiety symptoms are more persistent and irrelevant to positional changes. The diagnostic criteria for identifying POTS include: (1) a sustained increase in heart rate of at least 30 beats/min (bpm) in adults and at least 40 bpm in adolescents aged 12-19 years when transitioning from a supine to a standing position during 10-min standing test, (2) the absence of postural hypotension, which was defined as a decrease in systolic blood pressure exceeding 20 mmHg, and (3) the presence of symptoms indicative of postural intolerance for a minimum duration of 6 months (2–4).

The primary pathophysiological mechanisms underlying POTS include impaired sympathetic-mediated vasoconstriction in the lower extremities (i.e., neurogenic POTS), elevated cardiac sympathetic activity (i.e., hyperadrenergic POTS), dysregulation of blood volume, and reduced exercise capacity (5). Patients with neurogenic POTS may exhibit symptoms associated with neuropathy, such as sensory abnormalities and movement disorders. In contrast, patients with hyperadrenergic POTS may exhibit symptoms associated with hyperadrenergic activation, including palpitations, anxiety, and tremors. Furthermore, POTS may also be attributed to compromised cerebral blood flow autoregulation, genetic predispositions, upright hypertension, and psychological factors, such as anxiety, panic, and hypervigilance (2, 6). No pharmacological treatments specifically designed for POTS have demonstrated efficacy in ameliorating the condition. Consequently, the majority of patients necessitate an integrative approach comprising pharmacotherapy, lifestyle modifications, and physical therapy to mitigate the frequency of symptomatic episodes (7, 8). A comprehensive understanding of the underlying pathophysiological defects or dysfunctions is crucial for classifying POTS subtypes and guiding the development of targeted therapeutic interventions. Research showed that paroxetine has unique pharmacological and clinical properties compared with other selective serotonin reuptake inhibitors (SSRIs). It effectively alleviates symptoms of anxiety disorders, such as social anxiety disorder, with efficacy comparable to other SSRIs. Additionally, studies suggested that paroxetine may reduce all-cause and cardiovascular mortality after myocardial infarction (9–11). This case study details the administration of paroxetine in a patient hospitalized for recurrent syncope associated with POTS, with long-term follow-up indicating the treatment’s efficacy.

## Case presentation

### Primary hospitalization

The patient was a 17-year-old female student who was initially hospitalized following a 5-min episode of syncope. On October 28, 2023, at 15:00 h, she experienced dizziness and weakness during an 800-meter sprint as part of a school physical fitness test. This was followed by a loss of consciousness, syncope, and subsequent collapse to the ground. She regained consciousness spontaneously after approximately 5 min. Upon regaining consciousness, she experienced dizziness, the sensation of heaviness in the head, and generalized weakness. In 2020, the patient experienced transient syncope associated with sprinting. However, she was not admitted for inpatient care and consequently did not undergo a comprehensive diagnostic evaluation or receive appropriate treatment. The patient experienced dizziness during stressful situations or intense exercise. The patient had no known history of other medical conditions, no family history of cardiovascular diseases, and no documented allergies to medications or foods. Both general and neurological examinations revealed no significant abnormalities.

**Figure 1:**
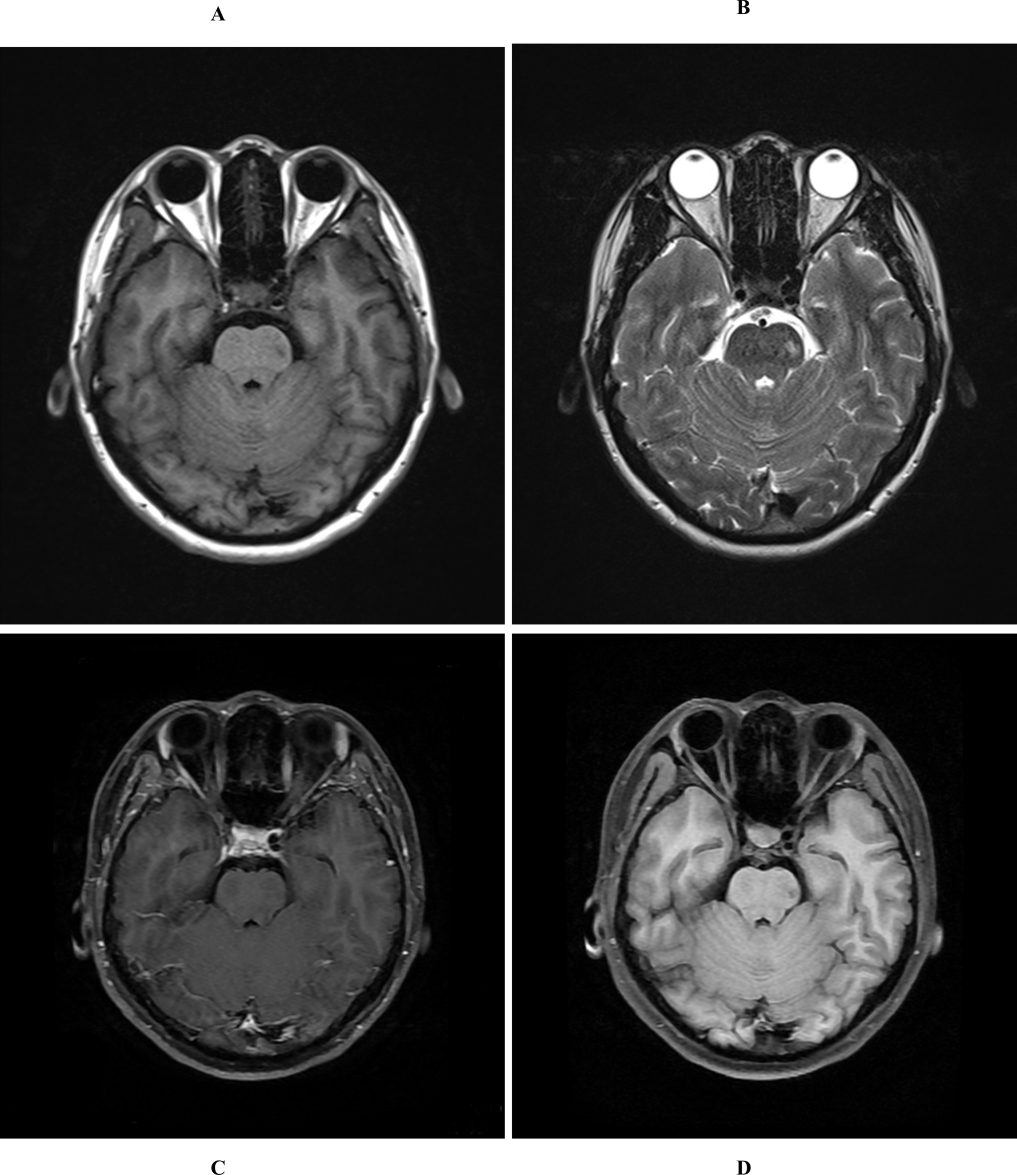
Cranial MRI of the patient. **(A)** T1-weighted imaging; **(B)** T2-weighted imaging; **(C)** The contrast-enhanced cranial MRI; **(D)** Fluid-attenuated inversion recovery (FLAIR) imaging.

Following admission, laboratory analyses revealed elevated levels of creatine kinase (1208.4 U/L) and creatine kinase isoenzyme (31 U/L), which could be attributable to the patient’s recent sprinting activity. Comprehensive evaluations were conducted, including blood, urine, and stool analyses, along with assessments of biochemical profiles, coagulation parameters, thyroid function, D-dimer levels, and N-terminal prohormone of brain natriuretic peptide (NT-proBNP) levels. These evaluations indicated no significant abnormalities. However, cranial magnetic resonance imaging (MRI) identified a patchy abnormal signal lesion measuring approximately 0.6 × 0.6 cm^2^ on the left side of the pons. On T1-weighted imaging (T1WI), it presented as low-signal intensity, whereas T2-weighted imaging (T2WI) and fluid-attenuated inversion recovery (FLAIR) sequences demonstrated high-signal intensity. Contrast-enhanced cranial MRI revealed no significant enhancement, indicating that the lesion is benign, an old infarct, or associated with demyelinating changes (**Figure 1**). Further diagnostic evaluations, including electrocardiography, transthoracic echocardiography, pulmonary function testing, and continuous electroencephalography revealed no significant abnormalities. On November 2, 2023, orthostatic test was conducted, revealing a heart rate of 73 bpm in the prone position and 126 bpm in the upright position. The change in blood pressure was less than 10 mmHg, accompanied by symptoms of dizziness, palpitations, trembling, blurred vision, and sensation of impending syncope. Collectively, these clinical findings satisfy the diagnostic criteria for POTS. Additional assessments were recommended, including an enhanced upright tilt test, head and neck computed tomography angiography (CTA), and continuous electrocardiographic monitoring. During hospitalization, the patient also presented with mutism and involuntary hand tremors, despite normal thyroid function. This raised suspicion of comorbid anxiety, warranting comprehensive psychiatric evaluation. However, the patient reported an improvement in symptoms and the absence of syncope episodes, leading to a request for discharge on the same day. Following comprehensive analysis of the patient’s condition, the initial diagnosis was identified as POTS, with the secondary diagnosis of a suspected anxiety disorder. Subsequently, the patient was discharged with specific instructions to consume 2-3 liters of fluids daily. Additionally, the patient was advised to engage in the development of a long-term, gradual, and regular exercise regimen, incorporating non-standing aerobic activities and resistance training targeting the thigh muscles.

### Secondary hospitalization

On the afternoon of November 9, 2023, the patient experienced a recurrence of syncope, presenting with symptoms identical to previous episodes, necessitating transport to our hospital by ambulance. Subsequently, the patient’s family requested a transfer to a local hospital for further treatment, resulting in 1-day hospitalization.

### Tertiary hospitalization

Following discharge from the hospital, the patient experienced over 10 syncopal episodes in a single day while in transit. The frequency of these syncopal events was markedly elevated compared with previous occurrences, involving a progressively worsening pattern of symptoms. Consequently, the patient was readmitted to our hospital on November 11, 2023. During this admission, the patient presented not only with recurrent syncope, but also with involuntary tremors that intensified with nervousness and were markedly more severe than in previous episodes. Additionally, the patient experienced generalized weakness, nausea, headache, dizziness, sensation of heaviness in the head, anxiety, restlessness, insomnia, and frequent awakenings. On this admission, in addition to aggressive blood volume supplementation, a small dose of propranolol (5 mg, orally) was given, which was immediately discontinued due to signs of intolerance, such as fatigue.

The complete cranial CTA of the head and neck displayed no significant abnormalities. In addition, dopamine, norepinephrine, and epinephrine in the blood were at normal levels. Furthermore, 24-h ambulatory electrocardiogram indicated the occurrence of seven supraventricular premature beats, with an average rhythm of 67 bpm, the fastest rhythm of 135 bpm (occurring at 16:05), and the slowest rhythm of 47 bpm (occurring at 04:02). The 24-h ambulatory blood pressure monitoring suggested a minimum diastolic blood pressure of 32 mmHg and a minimum systolic blood pressure of 81 mmHg throughout the day (**Table 1**). In addition, the psychological assessment was expanded to include the Hamilton Depression Rating Scale (HAMD, 16 points) (**Table 2**) and the Hamilton Anxiety Rating Scale (HAMA, 23 points) (**Table 3**). The diagnosis of anxiety disorder was made based on the criteria outlined in the DSM-5, following a comprehensive clinical assessment and standardized diagnostic interviews. Based on the established efficacy of paroxetine in treating anxiety disorders with autonomic dysfunction, paroxetine hydrochloride tablets (20 mg/day, orally) were administered as anxiolytic treatment. Starting on the third day of paroxetine treatment, the patient’s POTS gradually improved, and on the fifth day, syncope was significantly controlled. After one week of treatment with paroxetine, syncope did not recur in any of the subsequent sessions. In addition, psychogenic anxiety and somatization anxiety exhibited significant improvement, and she was discharged from the hospital.

**Table 1:** 24-h ambulatory blood pressure monitoring.

Period	Type	Average values	Maximum	Timing	Minimum	Timing	Blood pressure load	Average true variation (mmHg)
Daytime (07:00)	SBP	111	138	12:02:04	90	07:01:59	3.45	8. 46
	DBP	71	96	21:32:04	51	07:01:59	10.34%	9. 89
	Average	87	112	21:32:04	70	12:32:09		
	PR	71	111	08:34:14	55	07:01:59		
Night (22:00)	SBP	94	113	22:02:00	81	02:02:00	0.00%	9.88
	DBP	49	73	22:02:00	32	03:02:12	12.50%	10.63
	Average	65	89	22:02:00	49	02:02:00		
	PR	54	62	06:02:08	49	02:02:00		
Whole day	SBP	108	138	12:02:04	81	02:02:00	2.70	8.78
	DBP	66	96	21:32:04	32	03:02:12	10.81	10.06
	Average	82	112	21:32:04	49	02:02:00		
	PR	67	111	08:34:14	49	02:02:00		
Systolic blood pressure (mmHg): 111 (daytime), 94 (nighttime), with a difference of 17. The rate of decrease in blood pressure was 15.32%, and the limiting difference ratio (nighttime/daytime) was 66.67%.								
Diastolic blood pressure (mmHg) was 71 during the daytime, 49 at nighttime, with a difference of 22 mmHg, indicating a reduced blood pressure rate of 30.99%, and a limit difference ratio (night/day) of 91.11%.								
Differential pulse pressure (mmHg): 40 (daytime), 45 (evening), and 42 (whole-day).								
Systolic blood pressure greater than 140 mmHg: 0 occurrences, 0% frequency					Difference in systolic blood pressure greater than 30 mmHg:2 occurrences, 5.41% frequency			
Diastolic blood pressure greater than 90 mmHg: 1 occurrence, 2.7% frequency					Diastolic blood pressure difference greater than 15mmHg: 8 occurrences, 21.62% frequency			
Standard deviation: SBP = 12.92, DBP = 14.20					Variability: SBP = 0.12, DBP = 0.22			
Dynamic atherosclerosis index: -0.22					Trough-to-peak ratio: SBP = -0.50, DBP = 33.17			
Smoothing index: SBP = 0.03, DBP = 0.03					Morning peak blood pressure: SBP = 23.17, DBP = 33.17			

SBP, systolic blood pressure; DBP, diastolic blood pressure; PR, pulse rate.

**Table 2:** Hamilton Depression Rating Scale scores.

Item	Score	Item	Score
1. Depressed mood	0	13. Somatic symptoms, general	2
2. Feelings of guilty	1	14. Genital symptoms	0
3. Suicide	0	15. Hypochondriasis	0
4. Insomnia, early	2	16. Insight	0
5. Insomnia, middle	2	17. Loss of weight	0
6. Insomnia, late	2	18. Diurnal variation	1
7. Work and interests	0	19. Depersonalization or derealization	0
8. Psychomotor retardation	1	20. Paranoid symptoms	0
9. Psychomotor agitation	1	21. Compulsive symptoms	1
10. Anxiety, psychic	1	22. Helplessness	0
11. Anxiety, somatic	2	23. Hopelessness	0
12. Somatic symptoms, gastrointestinal	0	24. Worthlessness	0
Anxiety/somatization		0.83	
Weight		0.00	
Cognitive disturbance		0.50	
Diurnal variation		1.00	
Retardation		0.25	
Sleep disturbance		2.00	
Hopelessness		0.00	
total score		16	

Anxiety/somatization consists of six items: 10, 11, 12, 13, 15, and 17. Weight is represented by item 16. Cognitive disturbance includes six items: 2, 3, 9, 19, 20, and 21. Diurnal variation is represented by item 18. Retardation includes items 1, 7, 8, and 14. Sleep disturbance comprises items 4, 5, and 6. Hopelessness is composed of items 22, 23, and 24.

**Table 3:** Hamilton Anxiety Rating Scale scores.

Item	Score	Item	Score
1. Anxious mood	0	8. Sensory symptoms	2
2. Tension	3	9. Cardiovascular symptoms	2
3. Fears	0	10. Respiratory symptoms	1
4. Insomnia	3	11. Gastrointestinal symptoms	0
5. Intellectual (cognitive)	2	12. Genitourinary symptoms	0
6. Depressed mood	0	13. Autonomic symptoms	4
7. Muscular symptoms	3	14. Behavior at interview	3
Somatic anxiety	1.71	Psychic anxiety	1.57
Total score		23	

Somatic anxiety consists of seven items: 7, 8, 9, 10, 11, 12, and 13. Psychic anxiety comprises seven items: 1-6 and 14.

### Follow-up

**Figure 2:**
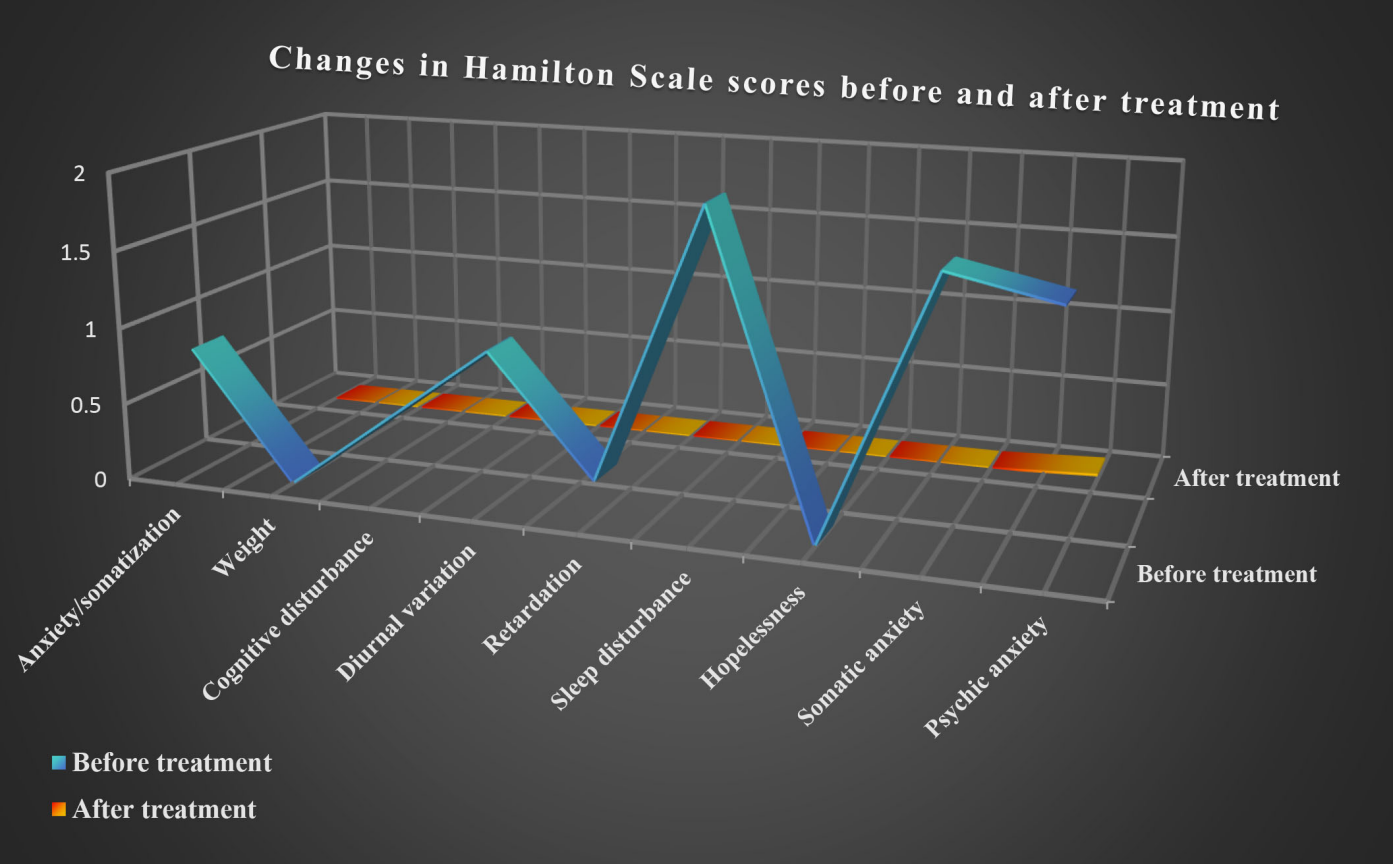
Changes in Hamilton Scale scores before and after treatment.

On December 28, 2023, the patient presented with symptoms, including chest tightness, dyspnea, dizziness accompanied by chills and tremors, auditory hallucinations (specifically abnormal bird sounds and whispering voices), and, in severe instances, sensation of choking. These symptoms persisted for approximately 15 min and subsequently resolved spontaneously, following an interpersonal conflict with a romantic partner. The dosage of paroxetine hydrochloride tablets was escalated to 40 mg per day, maintaining the same frequency of administration. Subsequently, the aforementioned symptoms progressively subsided. On May 16, 2024, a tapering regimen was initiated, in which the dosage was reduced by 10 mg every 17 days, leading to the complete discontinuation of paroxetine on July 6, 2024. In this study, the approach taken is based on a combination of standard protocols established in our clinical setting and individualized decisions tailored to each patient’s response. Subsequent assessments conducted on August 31, 2024 using the self-rating depression scale (SDS) score indicated no symptoms of depression and anxiety. On September 5, 2024, the upright test was re-administered and yielded a negative result. Additionally, the SCL-90 score was evaluated, with the Beck Anxiety Inventory (BAI), Beck Depression Inventory-21 (BDI-21), HAMD, and HAMA scores of 24.99, 2, 0, and 2 points, respectively. Upon subsequent follow-up, the patient experienced no further episodes of syncope following discontinuation of the medication. Additionally, symptoms of anxiety and depression were eliminated, and there was no recurrence of POTS (**Figure 2**).

## Discussion

This case study described a young woman who initially presented with a progressive exacerbation of syncope, accompanied by significant autonomic dysfunction. During her hospitalization, comprehensive diagnostic evaluations, including dynamic electroencephalogram, spirometry, NT-proBNP measurement, CTA of the head and neck, transthoracic echocardiography, and thyroid hormone assessments were conducted, revealing no significant abnormalities. Epilepsy, thyroid disease, cerebrovascular lesions, and cardiovascular diseases were excluded. Furthermore, 24-h ambulatory blood pressure monitoring and electrocardiography indicated only slightly low diastolic blood pressure and cardiac rhythm, while the upright test revealed significant positivity. Following the primary hospitalization, where blood volume expansion was combined with appropriate physiotherapy, no syncope recurred. However, during the patient’s secondary hospitalization, the condition significantly progressed, manifesting as frequent episodes of syncope. Due to the short-term hospitalization, no relevant treatment was administered. After discharge, the patient experienced approximately 20-30 episodes of syncope per day, along with persistent involuntary shaking, apprehension, insomnia, malaise, nausea, headache, dizziness, and sensation of heaviness in the head. Research indicated that propranolol is efficacious in alleviating anxiety symptoms at lower dosages (e.g., 5 mg), thereby reducing the likelihood of adverse effects typically associated with higher dosages (12). On the tertiary admission, a small dose of propranolol (5 mg, orally) was given in addition to aggressive blood volume expansion.

Hypovolemia is frequently identified in patients with POTS. Rapid restoration of blood volume through intravenous saline or pressor analogs may improve upright tolerance and alleviate symptoms related to heart rate variability during standing in patients with POTS (13–15). Low-dose propranolol reduces upright tachycardia and effectively improves symptoms in patients with POTS (2, 15, 16). However, in therapeutic observations, increasing blood volume had minimal impact on improving POTS symptoms or reducing the frequency of syncopal episodes in this patient. While propranolol slightly alleviated symptoms of autonomic dysfunction, such as hand tremors, it did not significantly improve POTS. Furthermore, the blood pressure reduction induced by propranolol exacerbated dizziness and increased the frequency of syncope, leading to its discontinuation.

Although no signs of anxiety or depression were initially noted during clinical evaluation, psychological assessment was conducted during the tertiary hospitalization due to the patient’s remarkable autonomic dysfunction symptoms. The HAMD scores indicated mild depression, primarily reflected in sleep disturbances and somatization anxiety. Symptoms included difficulty in falling asleep, disrupted sleep continuity, early awakening, hand tremors, dry mouth, dizziness, headache, hyperventilation, and chest tightness. The HAMA scores revealed significant anxiety, with elevated scores for somatic and mental anxiety factors. Symptoms included notable nervousness, sleep disturbances, muscle twitching, teeth chattering, voice trembling, generalized weakness, chest tightness, and palpitations, with autonomic dysfunction symptoms being most severe. Studies indicated that 87% of patients with POTS experienced mild-to-moderate anxiety and depression, and over half reported disturbed sleep patterns (17, 18). Additionally, anxiety and depression levels could be positively correlated with POTS symptom severity (19–21). These psychological factors further exacerbate POTS symptoms, complicating diagnosis and management (17, 22). While treating POTS may partially alleviate anxiety and depression, evidence on whether anxiolytic therapies can directly improve POTS symptoms remains limited (18, 20). According to the patient’s condition, treatment with paroxetine hydrochloride was initiated, resulting in a significant improvement in POTS symptoms and a rapid cessation of progressive syncope episodes.

The significant overlap between the somatic symptoms of anxiety and those of POTS can lead to the misdiagnosis of POTS as anxiety. This raises a critical question: can anxiolytic medications effectively treat POTS? In this case, the administration of paroxetine, an anxiolytic, led to a substantial improvement in POTS symptoms and cessation of progressive syncope episodes, suggesting two hypotheses. One hypothesis is that somatic anxiety symptoms may actually reflect underlying POTS pathology, and managing anxiety indirectly alleviates POTS symptoms. Based on this hypothesis, the severity of somatic anxiety symptoms prompted consideration of a comorbid anxiety disorder. Moreover, the patient’s somatic anxiety was significantly worse than psychogenic anxiety, aligning with the abovementioned hypothesis. Previous studies have also found that the majority of patients with POTS do not have excessive psychogenic anxiety although they exhibit significant somatization anxiety, confirming the abovementioned hypothesis (23). However, it has been demonstrated that POTS has unique cardiovascular features and pathophysiologic mechanisms, and it should not be diagnosed as anxiety due to some hyperadrenergic manifestations (24, 25). Although these studies confirm the existence of independent pathophysiologic mechanisms in POTS, there is currently no definitive evidence to exclude the possibility of anxiety co-occurring with POTS. The second hypothesis suggests that POTS and anxiety are distinct disorders, sharing a common pathological mechanism. Consequently, the use of anti-anxiety medications may influence the shared pathophysiological pathways, thereby exerting therapeutic effects on POTS. This hypothesis is supported by the observed case, where the patient experienced significant improvement in both POTS and anxiety symptoms following treatment with paroxetine. The literature review indicated that while plasma norepinephrine (NE) concentration, systemic NE spillover, plasma 3,4-dihydroxyphenylethanol (DHPG) concentration, and plasma renin activity (PRA) in patients with POTS were consistent with those of healthy individuals, POTS patients exhibit a significant reduction in norepinephrine transporter (NET) protein in peripheral sympathetic nerves and impaired synaptic norepinephrine clearance (26, 27). NET plays a crucial role in norepinephrine reuptake in synapses (28). Studies have shown that NET dysfunction is associated with major depressive disorder, panic disorder, anxiety disorders, POTS, and hypertension (29–31). In animal models of anxiety and depression, lower levels of SERT and NET were found in the hippocampus and brainstem. Paroxetine has been shown to alleviate anxiety and depression by increasing 5-HTT and NET levels (32). This suggests that NET dysfunction may represent a common pathological factor in both POTS and anxiety. Paroxetine may improve POTS symptoms while also exerting anxiolytic effects through modulation of NET expression and function. Although a temporal association between paroxetine initiation and symptom resolution was found, causality could not be definitively established in the absence of a controlled trial.

In addition, in the present study, contrast-enhanced cranial MRI revealed an infarct focus in the left pontine region, approximately 0.6 × 0.6 cm in size. Based on its characteristic features, this lesion was identified as a small deep pontine infarction (SDPI). Brainstem lesions may affect the functional connectivity of central autonomic networks. Studies have found that functional connectivity between the brainstem and cortical and subcortical structures is significantly reduced in patients with autonomic dysfunction. This diminished connectivity may lead to imbalances in autonomic regulation, which may trigger symptoms such as POTS (postural tachycardia syndrome) (33). SDPI may result from intrinsic lesions of the perforating arteries, such as lipohyalinosis or microatherosclerosis, typically presenting as a solitary lesion with a low likelihood of recurrence (34, 35). The clinical manifestations of SDPI may include pure motor syndrome, pure sensory syndrome, sensorimotor syndrome, ataxic hemiparesis, Dysarthria-Clumsiness syndrome, and Dysarthria-Clumsy hand syndrome, with pure sensory syndromes reported as the most frequent presentation (34). In this case, the patient exhibited symptoms, such as dizziness and syncope, but did not demonstrate significant ataxia or other neurological deficits. The results of clinical neurological tests, including the closed-eye-difficulty sign, finger-nose test, heel-knee-tibia test, and alternation test, were all normal, and no sensory or motor abnormalities were identified. Notably, a case with a similar pontine infarct location has been reported, in which the patient also lacked major neurological deficits and presented only with contralateral restless legs syndrome (36). This finding highlights the variability in clinical impact depending on the exact location and extent of pontine infarcts. The pons plays a crucial role in several neural functions, including regulation of the sleep-wake cycle, respiration, cardiovascular control, and vestibular reflexes. Additionally, it serves as a hub for neural pathways that mediate interactions between the vestibular system and the autonomic nervous system. A neural pathway connecting the vestibular nuclei to the nucleus tractus solitarius has been identified in the pons, and disruptions to this pathway can directly affect autonomic regulation, potentially resulting in abnormal heart rate and blood pressure responses similar to POTS (37). While it remains elusive whether the SDPI in this patient directly triggered POTS, the possibility of such a relationship could not be excluded. The lesion could theoretically disrupt neural signaling pathways, leading to autonomic dysfunction. However, considering the patient’s clinical history and subsequent treatment outcomes, it is more likely that the SDPI could be an isolated infarction resulting from inadequate perfusion of the deep perforating artery secondary to a POTS episode, rather than the primary cause of the condition. However, the link between pontine lesions and POTS is speculative, representing a theoretical contribution rather than a proven etiology.

This study has several limitations that should be taken into account when interpreting the findings. Firstly, as a single case report, its results cannot be applied to a wider patient population. Secondly, the study did not measure NET protein or SERT levels before and after treatment, limiting the ability to analyze the biological mechanisms involved in detail. Additionally, the absence of a control group indicates that the placebo effect or spontaneous remission cannot be ruled out, potentially impacting the accurate evaluation of treatment effectiveness. Lastly, the study lacked a blinded psychiatric assessment and formal DSM-5 diagnostic process, potentially introducing assessment bias and affecting diagnostic accuracy. Future research should confirm these preliminary findings through a multicenter, large-sample, randomized controlled trial with dynamic biomarker monitoring and a standardized diagnostic approach.

## Conclusions

It is essential for physicians to recognize that the diagnostic criteria for POTS define a clinically acknowledged condition, making timely and accurate diagnosis critical. The pathogenesis of POTS remains incompletely understood, and diagnosis is primarily based on symptomatic presentation and clinical signs due to the lack of specific biomarkers. In this case, treatment with paroxetine was temporally associated with a reduction in progressive syncopal episodes. This suggests the possibility that POTS and anxiety disorders may share overlapping pathophysiological mechanisms, such as dysfunction of norepinephrine transport. Paroxetine may exert its therapeutic effects by targeting these shared pathways, thereby improving both POTS and anxiety symptoms. Notably, this is one of the few documented cases where POTS symptoms with comorbid anxiety responded positively to SSRI monotherapy, highlighting the necessity of further research into SSRIs as potential adjunctive treatments for POTS. However, given that this conclusion is based on a single case, broader generalizations should be made cautiously. Additionally, prospective trials evaluating SSRIs in POTS patients with comorbid anxiety are warranted. The NET may play a remarkable role in understanding how paroxetine concurrently addresses both conditions by modulating underlying pathophysiological pathways. Future studies should utilize a randomized, double-blind, placebo-controlled trial design to assess the efficacy of paroxetine compared with placebo in POTS treatment. Moreover, further research is necessary to elucidate the pathophysiological mechanisms linking POTS with anxiety and to validate the potential role of NET as a therapeutic target.

## Data Availability

The raw data supporting the conclusions of this article will be made available by the authors, without undue reservation.
